# Restorative therapy using microglial depletion and repopulation for central nervous system injuries and diseases

**DOI:** 10.3389/fimmu.2022.969127

**Published:** 2022-07-14

**Authors:** Weipeng Shi, Jing Zhang, Zhen Shang, Yingze Zhang, Yanzhi Xia, Haitao Fu, Tengbo Yu

**Affiliations:** ^1^ Department of Sports Medicine, The Affiliated Hospital of Qingdao University, Qingdao University, Qingdao, China; ^2^ Medical Department of Qingdao University, Qingdao, China; ^3^ Key Laboratory of Biomechanics of Hebei Province, Department of Trauma Emergency Center, The Third Hospital of Hebei Medical University, Orthopaedics Research Institution of Hebei Province, Shijiazhuang, China; ^4^ State Key Laboratory of Bio-Fibers and Eco-Textiles, Collaborative Innovation Center for Marine Biomass Fibers, Materials and Textiles of Shandong Province, College of Materials Science and Engineering, Institute of Marine Biobased Materials, Qingdao University, Qingdao, China

**Keywords:** microglia, depletion, repopulation, central nervous system, diseases

## Abstract

Microglia are important resident immune cells in the central nervous system (CNS) and play an important role in its development, homeostasis, and disease treatments. Activated microglia perform diverse functions in mouse models of CNS neurodegenerative diseases or deficits. In humans, microglia have been linked to various neurodegenerative diseases. Following brain or spinal cord injury, microglia express pro- and anti-inflammatory phenotypes at different stages of recovery. With the development of pharmacological and genetic tools for microglial depletion, studies have demonstrated that microglial depletion exerts both positive and negative effects in the treatment of CNS diseases. Notably, microglial depletion provides an empty niche that stimulates production of new microglia. Microglial depletion and repopulation can not only treat diseases by eliminating dysfunctional microglia but can also provide an indication of the molecular mechanisms of diseases. Although this approach has shown impressive results, its use is still in its infancy. In this review, we summarize the current pharmacological and genetic tools for microglial depletion and highlight recent advances in microglial repopulation therapy for the treatment and functional recovery of neurological diseases and deficits. Finally, we briefly discuss the therapeutic challenges and prospective uses of microglial repopulation therapy.

## Introduction

Microglia are resident macrophages in the central nervous system (CNS), which are derived from the yolk sac progenitor cells and migrate to the CNS between embryonic day 8.5 and time of formation of the blood-brain barrier (BBB) (1, 2). Microglia play different roles in the CNS according to the development and maturation stage of the CNS. For example, synaptic pruning of the microglia plays an important role in the formation of neural circuits during CNS development (3) and removal of dead cells and myelin debris in pathological conditions. Microglia also drive neuronal programmed cell death by inducing neuronal apoptosis, thereby eliminating excess neurons produced during development (4). In addition, microglial protrusions monitor the surrounding environment and directly communicate with neurons, astrocytes, and blood vessels (5). Based on the surveillance of surrounding information, microglia can transduce a variety of extracellular signals to maintain a stable environment in the brain (3). Microglia rapidly react to changes in the CNS microenvironment (e.g., infection, trauma, and disease) by changing from the resting state to the active state, to remove tissue debris and restore homeostasis (6, 7).

Initially, activated microglia are divided into M1- and M2-like phenotypes (8). M1 microglia play a key role in the host immune response by producing pro-inflammatory cytokines (e.g., tumor necrosis factor alpha [TNF-α] and interleukin [IL]-6), inducible nitric oxide synthase (iNOS), and reactive oxygen species (ROS) (9, 10). M2 microglia produce anti-inflammatory cytokines, chemokines, and growth factors, which inhibit the inflammatory response and promote tissue repair (11, 12). However, it is still controversial that M1 and M2 phenotypes exist *in vivo* (13). With the development of technologies such as single-cell RNA sequencing (scRNA-seq), scholars have gradually realized that microglia are highly heterogeneous (14), “M1” and “M2” may not reflect microglial states precisely. Several studies have demonstrated that microglial hyperactivation and dysregulation lead to neurotoxicity, which are associated with neurodegenerative diseases, including epilepsy (15), Alzheimer’s disease (AD) (16), Parkinson’s disease (PD) (17), and Huntington’s disease (18). The microglial response to injury is partly regulated by interactions with other glial cells (19). For example, in spinal cord injury (SCI) models, activated microglia and reactive astrocytes interact to form glial scars, which impact neuronal and functional recovery (20). Meanwhile, TNF-α expression by activated microglia contributes to astrocyte glutamate production and leads to neuronal excitotoxicity (21). In the meantime, TNF-α induces activated microglia to release glutamate and produce neurotoxicity through autocrine manner (22). In addition, the products released by activated microglia, including glutamate, nitric oxide (NO), IL-1b, and TNF-a, promote oligodendrocyte death (23, 24). Microglia-mediated inflammation is a significant contributor to the microenvironment after CNS injury and diseases, which persists for a long duration and affects CNS repair (25). Therefore, depleting the hyperactivated microglia may be an effective approach to treat CNS diseases and injury.

Microglial depletion from the CNS microenvironment has been extensively studied as a treatment for CNS diseases, and has dual effects on CNS diseases and deficits. In recent years, microglial depletion and repopulation have received significant attention. In the present review, we describe approaches for microglial depletion and the source and dynamics of microglial repopulation based on recent studies. In addition, we summarize current research on microglial repopulation for the treatment of CNS diseases or deficits, and briefly discusses the challenges and potential uses of microglial repopulation therapy.

## Microglial depletion for the treatment of CNS injuries and diseases

### Approaches for microglial depletion

**Figure 1:**
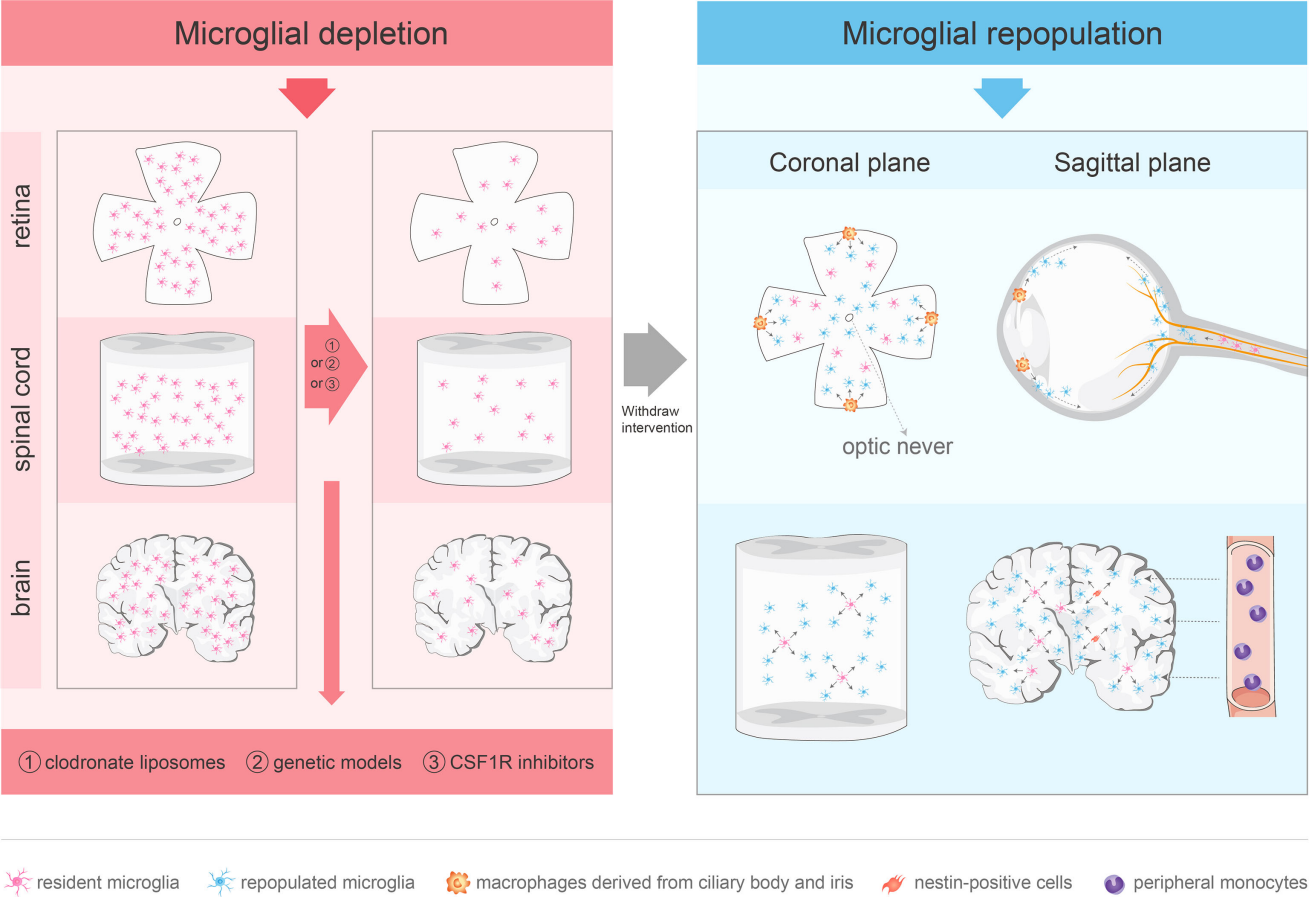
Approaches for microglial depletion and multiple sources of microglial repopulation. Robust methods for depleting microglia in vivo include clodronate liposomes, genetic models and CSF1R inhibitors. These methods deplete microglia in the CNS effectively. After withdraw the intervention, microglia repopulate and return to normal levels in 1–2 weeks. The repopulated microglia in the retina are not derived from nestin-positive progenitor cells. The repopulated microglia in the center arise from the residual microglia in the optic nerve, while the periphery-emerging microglia are from macrophages in the ciliary body/iris. Spinal microglia can reproduce rapidly after removal, which is mainly driven by the process of self-renewal. Microglia in the brain may repopulate in one of three ways: stimulation of microglia progenitor cells that express nestin, proliferation of residual microglia, or infiltration of peripheral mononuclear cells.

To investigate the role of microglial depletion in the treatment of CNS diseases, various depletion methods have been developed, including clodronate liposomes, genetic models, and colony-stimulating factor 1 receptor (CSF1R) inhibitors. (**Figure 1**).

Clodronate liposomes cannot cross the BBB or blood-retina barrier (BRB) and need to be injected directly into the CNS or vitreous to target macrophages and trigger their apoptosis without genetic modification (26–28). This approach is effective with rapid but short-lived effects. A recent study reported that clodronate liposomes cause microglial ablation in the mouse striatum at day 1, which persists for 3 days, and is followed by microglial reappearance after 5 days (29). However, this approach may damage the CNS immune privilege (IP) and lead to off-target effects (30).

Herpes simplex virus-derived thymidine kinase (HSVTK), a suicide gene, can convert the antiviral nucleotide analog prodrug, ganciclovir (GCV), into a monophosphate form, which is converted to a toxic triphosphate (30, 31). CD11b-HSVTK transgenic mice overexpress HSVTK driven by the Cd11b promoter. HSVTK can induce apoptosis in CD11b+ myeloid cells in the presence of GCV (32). After 2 weeks of GCV treatment, the absolute microglial number was reduced by 90%; a higher level of microglial depletion (> 95%) was achieved after 4 weeks of GCV treatment (33, 34). However, long-term GCV administration may lead to myelotoxicity because of the elimination of CD11+ cells and red blood cells (30, 35). Another novel lineage ablation system is based on the transgenic expression of diphtheria toxin receptor (DTR) in mouse cells and the application of diphtheria toxin (DT) (36). By crossing the inducible DTR (iDTR) strain with tissue-specific Cre-expressing mouse strain, the STOP cassette that prevents DTR expression was removed, which would lead to cell death of DTR-expressing myeloid cells in response to DT injection (36). Cx3cr1CreER:iDTR system ablates microglia following injections of tamoxifen (TAM) and DT (37). This system was associated with 80% depletion in the number of microglia 3 days after DT injection, which was followed by normalization of the microglia number at day 14 (37). However, this model also produced a strong neuroinflammatory response, termed a cytokine storm (37). Iba1-tTA::DTA^tetO/tetO^ mice also use the DT depletion system. DTA was selectively overexpressed in Iba1+ microglia after withdrawal of doxycycline (DOX), which led to a reduction in the number of retinal microglia by about 90% (38). The new microglia Cre lines (e.g., TMEM119) may be used to rapidly identify microglia (39). Similarly, the DT receptor gene expressed in the Cx3cr1 promoter of the Cx3cr1-Dtr transgenic rat model was associated with acute microglial ablation after DT application (40). An advantage of the TAM-induced Cre system is its selective targeting of microglia, but not of the circulating monocytes or other short-lived myeloid cells (37). The administration of TAM to Cx3r1CreER/+R26DTA/+ mice resulted in loss of approximately 95% of microglia on day 7 (41). In addition, Cre expression was lost over time in short-lived CX3CR1+ myeloid cells compared to the long-lived and self-renewing microglia (42, 43). However, most of genetic tools for microglia depletion target all macrophages and cannot accurately differentiate between microglia and CNS border associated macrophages (CAMs or BAMs), so CAMs or BAMs would also be affected (44). the valuable new genetic systems (e.g., Sall1, Siglec-H, and Hexb) can deplete microglia more accurately (45–47). In another Cx3cr1CreERT2/+-Csf1r+/fl system, the deletion of the Csf1r allele disrupts microglial homeostasis (48).

CSF1R is a critical regulator of microglial development and survival (49–53), and is expressed in microglia (26–28), neural progenitor cells (54), and several neuronal subpopulations in the CNS (54). In rodents, CSF1R inhibition causes a significant reduction in resident microglia. PLX3397 and PLX5622 are the most commonly used CSF1R inhibitors for microglia depletion, these CSF1R inhibitors can cross the BBB and deplete the microglia in the brain, spinal cord, and retina within a few days (49, 55, 56). The limitations of PLX3397 mainly include relatively low penetrance and potential off target effect (49, 57), while PLX5622 has higher CSF1R specificity and brain penetrance (58). However, Hohsfield et al. found high dose PLX3397 has higher microglial depletion efficiency (59). The microglial depletion is caused by microglia death rather than downregulation of microglia markers (58). Currently, the CSF1R inhibitors are widely used in mouse models of various CNS diseases or injuries (55, 58, 60–64). In general, most microglia were depleted 1–2 weeks after CSF1R inhibitors application, and microglia returned to the baseline level about 2 weeks after discontinuation of the CSF1R inhibitors. Notably, CSF1R inhibitors lead to continuous microglial depletion. However, the inhibitors are not specific for CSF1R and also inhibit other kinases (65, 66). In addition, treatment with CSF1R inhibitors may lead to broad myelosuppression (67), and microglial progenitors in bone marrow and tissue macrophages may be similarly affected (68, 69).

### Effects of microglial depletion on CNS injuries and diseases

Microglial depletion may have dual effects on CNS degenerative and traumatic diseases. For example, in AD mouse models, microglial inhibition prevents motor neuron degeneration and improves the function and cognition in mice (55, 70). In PD mouse models, CSF1R inhibition can attenuate cognitive deficits, neuronal damage, astroglial activation, proinflammatory factor production, and oxidative stress (71). Microglial inhibition enhances central remyelination and prevents demyelination in multiple sclerosis mice by modulating neuroinflammation (72). Transient microglial inhibition after SCI and traumatic brain injury (TBI) promotes motor and neurological recovery, improves depression-like behavior, prevents tissue damage, and downregulates the levels of proliferation-associated transcripts and inflammation-associated genes (73–77). CSF1R inhibitors plays a neuroprotective role in cerebral ischemic stroke mice by inhibiting microglial polarization and inflammatory pathway activation (78). In addition, microglial inhibiting reduces age-associated neuroinflammation in aging mouse brains (79). However, microglial inhibition may be detrimental to disease recovery in some cases. In animal models of CNS degenerative diseases, microglial depletion exacerbates the severity of KA-induced acute/chronic seizures (80), significantly increases inflammation, demyelination, and axonal degeneration in mice with experimental autoimmune encephalomyelitis (EAE) (81), and leads to increased plaque size over 1 week in AD mice (82). In contrast to the short-term microglial inhibition after SCI, prolonged microglial inhibition do not improve motor recovery (83), and even aggravates damage, reduces neuronal numbers, exacerbates axonal dieback, and hinders motor recovery (60). Moreover, microglial depletion reduces the clearance of degenerating neurons after TBI in pediatric rats (84) and leads to a significant increase in infarct size in post-stroke brain injury (64). Microglial depletion also affects retinal regeneration in zebrafish following retinal injury (85). Therefore, the outcomes of microglial depletion in the treatment of CNS diseases or deficits remains controversial, and the duration of depletion may influence the treatment results.

## Source and dynamics of repopulating microglia

### Multiple sources of microglial repopulation

In the normal adult mouse and human brain, microglia have long lives at the population level (86) and, at the individual cell level, they maintain dynamic stability by temporally and spatially coupling proliferation and apoptosis (87). After removal of the depleting tools, microglia may repopulate in one of three ways to return rapidly to the normal levels within 1–2 weeks: proliferation of residual microglia (87), stimulation of microglial progenitor cells in the adult brain (49), and infiltration by peripheral mononuclear cells (88).

PLX3397 treatment of CX3CR1-GFP+/− mouse model for 7 days demonstrated that the repopulated microglia were strongly nestin+, and Western blotting of whole brain homogenates showed a significant increase in nestin levels at day 3 of recovery. The repopulated microglia expressed nestin immunoreactivity and rapidly differentiated into branched microglia within 7–14 days. Importantly, the repopulated brain microglia induce proliferation of nestin-expressing cells throughout the CNS (49). Thus, Elmore et al. (49) suggested that the repopulating microglia are mainly derived from brain microglial progenitor cells that express nestin. However, a study based on fate mapping found that all the regenerated microglia originated from residual microglia, rather than nestin+ cells (89). Microglia transiently express nestin during regeneration and development, which may be a feature of microglial proliferation. Microglial regeneration demonstrates rapid proliferation kinetics and, after removal of CSF1R inhibition, the residual microglia proliferate rapidly and supplement the whole brain (56). Under physiological circumstances, microglia are separated from the peripheral circulatory system through the BBB and maintain their number through local proliferation without supplementation by blood monocytes (87, 90). Based on the macrophage niche theory (91), administration of selective CSF1R inhibitors may consume most microglia to deplete the microglial niche, which may trigger microglial reproliferation to return the microglial number to the baseline level (49, 92). Blood-derived monocytes have the potential to occupy the microglial niche in the adult CNS and repopulate the brain within 2 weeks. The newly engrafted myeloid cells have analogous functions to microglia (37).

Similar to brain microglia, spinal microglia can reproduce rapidly after being removed, which is mainly driven by the process of self-renewal. Although circulating monocyte infiltration was observed, the infiltration was part of the acute inflammatory response caused by cell death, which depends on MCP-1/CCR2 signal transduction (93). One of the main strategies of spinal cord microglial regeneration is similar to that for brain microglia, which proliferate by triggering the division of remaining cells (37, 56, 94), known as compensatory proliferation. Another strategy of spinal cord microglial regeneration is through compensatory cell hypertrophy (CCH), which is regulated by the insulin/insulin-like growth factor (IGF) signaling pathway (95). Notably, during spinal microglial regeneration, CCH occurs before cell proliferation (93).

Although blood cells in the diseased retina can differentiate into microglia (96, 97), the repopulated microglia in the center are only derived from the residual microglia in the optic nerve, while the peripheral microglia are produced by the macrophages in the ciliary body/iris. Microglia remaining in the optic nerve migrate to the retina along the center-to-periphery axis to repopulate the retina. Macrophages in the ciliary body relocate to the peripheral retina and migrate toward the central retina, whereas macrophages in the iris migrate to the peripheral retina through the ciliary body (56). However, the physiological and pathological roles of microglial radial migration are unknown. A previous study provided novel evidence that CX3CL1-CX3CR1 signaling regulates microglial regeneration kinetics in the retina by enhancing the proliferation and morphological maturation of regenerated cells (98). In addition, although the loss of P2Y12 affected microglial branching (99), P2Y12 receptor did not regulate the maturation of the newly produced microglia or affect doublet formation and repopulation. P2Y12 deletion impacted microglial morphology during repopulation but played a minor role in microglial division (99).(**Figure 1**)

### Mechanism of microglial regeneration

Microglia restore homeostasis during repopulation through self-renewal, proximity clonal expansion, and activation of maturation programs (100). As described previously, microglia can be regenerated almost entirely by self-renewal (37, 56); the microglial transformation of nestin+ progenitor cells were not detected under steady-state conditions (87). Similar to the clonal expansion of microglia in response to facial nerve injury (101), microglia proliferate through clonal expansion after ablation. Newborn microglia recolonize the parenchyma from the proximal clonal expansion to form unique spatial clusters and maintain a stable territorial boundary over time. Once microglial colonies are formed, they remain stable in the CNS (100). Recent studies have shown that inflammatory signals are vital for the regulation of microglial maturation and function (102, 103). Newborn microglia undergo a series of transcriptional changes and eventually transform from an immature state to a stable mature phenotype. The maturation process involves several steps, including the activation of nuclear factor kappa-light-chain-enhancer of activated B cells (NF-κB). NF-κB signaling enhances the early stages of microglia reproliferation (100). In addition, TGF-β signaling is important for microglial development and homeostasis (104) The microglial density in the brain is extremely stable (87), and microglia can return to the original homeostatic density through steady turnover even after acute ablation. Contact inhibition is one of the methods by which the microglia memorize their homeostatic density (100) Residual microglia repopulate freely by losing contact inhibition. For example, Syndecan4, a regulator of contact inhibition (105), is expressed at low levels 1 month after microglial repopulation (98). Moreover, in the CD11b-HSVTK transgenic mice model, blood-derived monocytes infiltrate and engraft in the brain of microglia-depleted mice, and maintain the myeloid component in the mature brain through homeostasis (88). A recently published paper of CNS myeloid cells under PLX3397 inhibition found that the MAC2^+^ microglial subpopulation in residual microglia is similar to microglial progenitors during development and could promote repopulation of microglia in the brain (106). In addition, multiple microglia subtypes, including white matter-associated microglia (WAM), have been identified by scRNA-seq (107). The recent study revealed a subpopulation of myeloid cells which derive from the subventricular zone and white matter, the population reconstitute microglia in the brain through a dynamic wave and exhibit similar the transcriptional profiles similar to disease-associated microglia (DAM) (59).

## Microglial repopulation therapy: A new treatment option for neurological diseases and deficits

**Figure 2:**
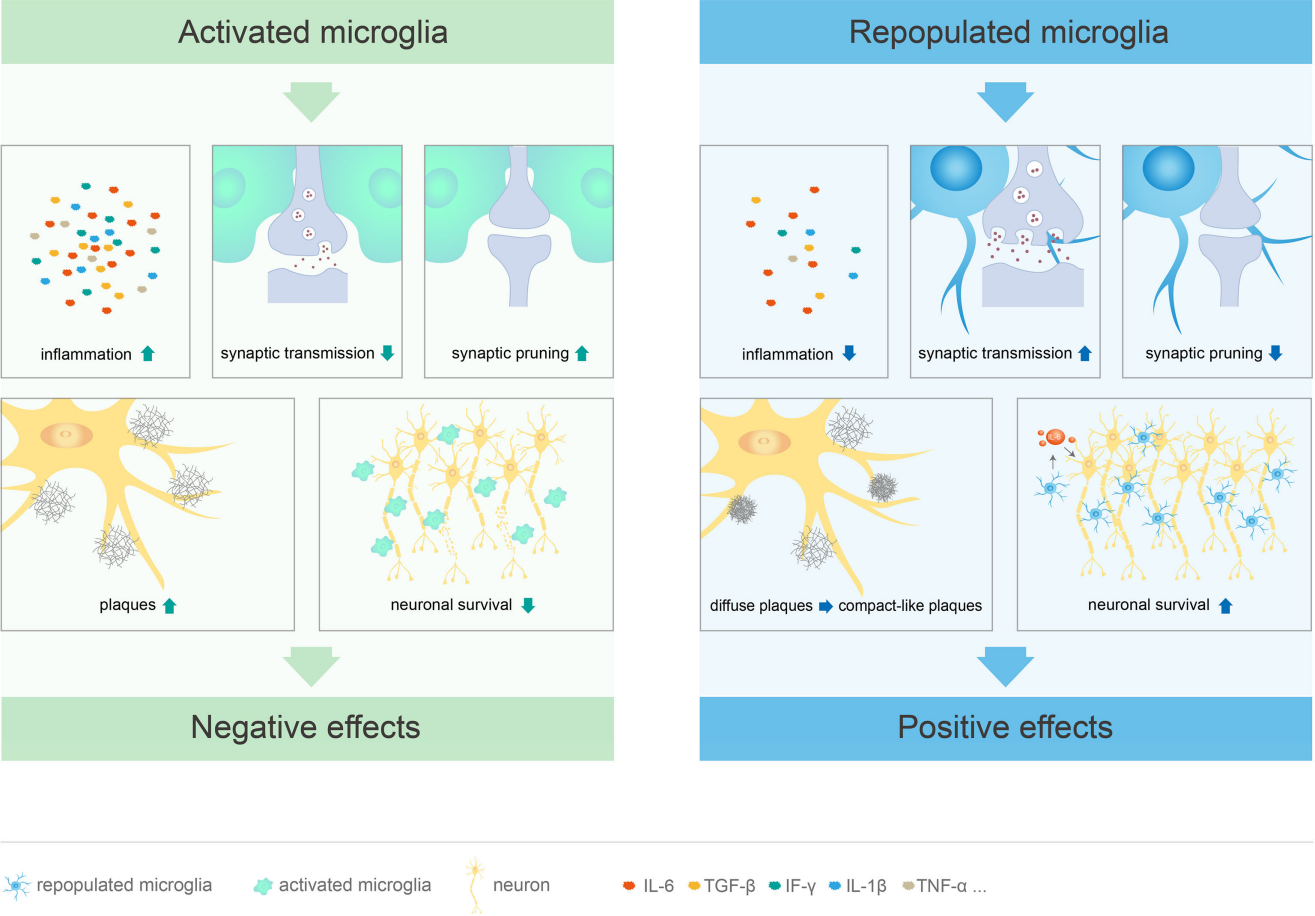
Different functions of activated and repopulated microglia in CNS diseases. Microglial activation leads to cognitive deficits through excessive synaptic pruning and inhibition of synaptic transmission. At the same time, these cells promote the release of inflammatory factors, stimulate plaque formation, and directly damage neurons, which leads to CNS diseases. Repopulated microglia not only suppress the inflammatory response, but also limit neuritic dystrophy by promoting the transition from diffuse to compact-like plaques. These cells can restore cognitive function by promoting synaptic transmission and inhibiting excessive synaptic pruning. Furthermore, repopulated microglia mediate neuroprotective effects by inducing IL-6 within neurons.

After pharmacological or genetically targeted depletion in healthy adult mice, microglia can repopulate in a short period of time by self-renewal (29, 49, 88) to restore to the baseline level and re-establish typical spine density (49, 100, 108). In addition, inflammation-related genes of repopulated microglia remained stable during the repopulation processes, without significant up- or down-regulation (56). Compared to the resident microglia, repopulated microglia exhibit distinct morphological features, with larger cell bodies and less complex branches formed in response to inhibitor removal; the morphological characteristics and synaptic function of the repopulated microglia gradually return to those of typical microglia after 4 weeks (50). Although the repopulated microglia are numerically and morphologically different from the resident microglia, they have similar mRNA levels, perform similar functions, do not affect behavior, cognition, or motor function, and do not have any side effects (50, 56, 88). Furthermore, microglial elimination and repopulation at a young age may have a more significant impact on behavior and cognition than in adults (50) Therefore, microglial repopulation therapy may be a potentially effective treatment for neurological diseases and deficits (**Figure 2**, **Table 1**).

**Table 1:** The outcomes of microglial repopulation in different CNS disease and deficits.

Depletion ways	Diseases or disorders	Outcomes	References
CX3CR1-iDTR+DT	AD	Repopulated microglia are associated with the stabilization of plaque size during the second week.	Microglia limit the expansion of β-amyloid plaques in a mouse model of Alzheimer’s disease (2017).
PLX5622	AD	Repopulated microglia result in more compact plaques predominating microglia-repopulated regions and execute disease-mitigating functions.	Microglia depletion rapidly and reversibly alters amyloid pathology by modification of plaque compaction and morphologies (2020)
PLX3397	AD	Repopulated microglia cluster around plaques, they have a reduction in disease-associated microglia (DAM) gene expression and elevate tau seeding/spreading.	Activated microglia mitigate Aβ-associated tau seeding and spreading (2021)
PLX3397	PD	Microglial repopulation could bring about apparent resistance to MPTP intoxication, and microglial replenishment elicits neuroprotection in PD mice.	Partial depletion and repopulation of microglia have different effects in the acute MPTP mouse model of Parkinson’s disease (2021)
PLX5622	Brain trauma	Repopulated microglia could resolve the proinflammatory response, promote functional recovery after brain injury, downregulate the expression of reactive microglial markers and reduce the levels of inflammatory-related genes.	Rice Rachel A,Pham Jason,Lee Rafael J et al. Microglial repopulation resolves inflammation and promotes brain recovery after injury.[J].Glia, 2017, 65: 931-944.
PLX5622	TBI	Repopulated microglia could improve neurological function, suppress neuroinflammatory and oxidative stress pathways, and reduce persistent neurodegenerative processes	Henry Rebecca J,Ritzel Rodney M,Barrett James P et al. Microglial Depletion with CSF1R Inhibitor During Chronic Phase of Experimental Traumatic Brain Injury Reduces Neurodegeneration and Neurological Deficits.[J].J Neurosci, 2020, 40: 2960-2974.
PLX5562/CX_3_CR_1_cre^ERT2^xiDTR	TBI	Repopulating microglia can attenuate learning deficits and stimulate neurogenesis, positively modulate the microenvironment of the injured brain and induce IL-6 in neurons and mediate neuroprotection.	Willis Emily F,MacDonald Kelli P A,Nguyen Quan H et al. Repopulating Microglia Promote Brain Repair in an IL-6-Dependent Manner.[J].Cell, 2020, 180: 833-846.e16.
PLX5622	Brain trauma	Microglial depletion and repopulation prevent radiation−induced hippocampal−dependent memory deficits, radiation−induced loss of hippocampal PSD−95 and eliminates radiation−induced transcriptome signatures	Feng Xi,Frias Elma S,Paladini Maria S et al. Functional role of brain-engrafted macrophages against brain injuries.[J].J Neuroinflammation, 2021, 18: 232.
PLX3397	SCI	Microglial/macrophage depletion and repopulation in combination with gelatin hydrogel transplantation resolves acute and chronic pro-inflammation, promotes endogenous neural stem/progenitor cell migration and neurogenesis and improves electrophysiological and functional recovery.	Ma Dezun,Zhao Yannan,Huang Lei et al. A novel hydrogel-based treatment for complete transection spinal cord injury repair is driven by microglia/macrophages repopulation.[J].Biomaterials, 2020, 237: 119830.
PLX5622	Aging	Repopulated microglia could reverse cognitive, synaptic, and neuronal deficits in the aged brain.	Elmore Monica R P,Hohsfield Lindsay A,Kramár Enikö A et al. Replacement of microglia in the aged brain reverses cognitive, synaptic, and neuronal deficits in mice.[J].Aging Cell, 2018, 17: e12832.
PLX5622	Aging	Microglial repopulation reduced CD68 expression, cleared lipofuscin, and partially restored the microglial RNA signature. However, priming and immune reactivity in the microglia of aged mice was not reversed by forcing microglial turnover.	O’Neil Shane M,Witcher Kristina G,McKim Daniel B et al. Forced turnover of aged microglia induces an intermediate phenotype but does not rebalance CNS environmental cues driving priming to immune challenge.[J].Acta Neuropathol Commun, 2018, 6: 129.
PLX3397	Aging	Microglial repopulation is associated with recovery of synaptic transmission and memory; however, repopulation do not rejuvenate synaptic transmission or cognitive function of aged animals to mirror a “younger” phenotype.	Yegla Brittney,Boles Jake,Kumar Ashok et al. Partial microglial depletion is associated with impaired hippocampal synaptic and cognitive function in young and aged rats.[J].Glia, 2021, 69: 1494-1514.
Cx3cr1-Dtr	Short-term memory	Microglia repopulate the brain after depletion, learning and memory performance is improved	De Luca Simone N,Soch Alita,Sominsky Luba et al. Glial remodeling enhances short-term memory performance in Wistar rats.[J].J Neuroinflammation, 2020, 17: 52.
PLX5622	Cognitive deficits	Repopulated microglia can improve cognitive deficits caused by cosmic radiation exposure	Krukowski Karen,Feng Xi,Paladini Maria Serena et al. Temporary microglia-depletion after cosmic radiation modifies phagocytic activity and prevents cognitive deficits.[J].Sci Rep, 2018, 8: 7857.
PLX5622	Repeated social defeat (RSD)	Microglial depletion and repopulation in RSD-sensitized mice do not affect hyperactivity under acute stress, but it effectively turnovers microglial reactivity to LPS challenge.	Weber Michael D,McKim Daniel B,Niraula Anzela et al. The Influence of Microglial Elimination and Repopulation on Stress Sensitization Induced by Repeated Social Defeat.[J].Biol Psychiatry, 2019, 85: 667-678.
PLX5622	Chronic social defeat (CSD)	Microglial repopulation of the brain post-CSD reintroduces adverse stress effects and leads to behavioral deficits.	Lehmann Michael L,Weigel Thaddeus K,Poffenberger Chelsie N et al. The Behavioral Sequelae of Social Defeat Require Microglia and Are Driven by Oxidative Stress in Mice.[J].J Neurosci, 2019, 39: 5594-5605.
PLX3397	Alcohol use disorders (AUDs)	Microglial depletion and repopulation can reverse chronic neuroimmune activation by normalizing proinflammatory cytokine levels and increasing protective trophic factors	Coleman Leon G,Zou Jian,Crews Fulton T,Microglial depletion and repopulation in brain slice culture normalizes sensitized proinflammatory signaling.[J].J Neuroinflammation, 2020, 17: 27.
CX3CR1-Cre^ERT2+/–^iDTR^+/–^	Experimental autoimmune encephalomyelitis (EAE)	Depletion and repopulation of microglia did not affect EAE neuropathology or CNS T-cell responses.	Rubino Stephen J,Mayo Lior,Wimmer Isabella et al. Acute microglia ablation induces neurodegeneration in the somatosensory system.[J].Nat Commun, 2018, 9: 4578.
BLZ945	Auditory brainstem deficits	Repopulated microglia can rectify anatomic defects and partially restore auditory function.	Milinkeviciute Giedre,Chokr Sima M,Cramer Karina S,Auditory Brainstem Deficits from Early Treatment with a CSF1R Inhibitor Largely Recover with Microglial Repopulation.[J].eNeuro, 2021, 8: undefined.
PLX3397	Fear-related disorders	Repopulated microglia contribute to eradicate fear memory in the mice of fear conditioning.	Cui Xiaoyu,Zhou Songhua,Xia Guang et al. A multispecies probiotic accelerates fear extinction and inhibits relapse in mice: Role of microglia.[J].Neuropharmacology, 2021, 193: 108613.
PLX3397	Intracerebral hemorrhage (ICH)	Repopulated microglia can reduce neuroinflammation, neurological deficits and brain edema following ICH in the aged brain.	Li Xiuping,Gao Xiaolin,Zhang Wenyan et al. Microglial replacement in the aged brain restricts neuroinflammation following intracerebral hemorrhage.[J].Cell Death Dis, 2022, 13: 33.

### Microglial repopulation therapy for neurodegenerative diseases

Regenerated microglia are beneficial for CNS neurodegenerative diseases, including AD and PD. AD is characterized by extracellular amyloid-β (Aβ) plaques and neurofibrillary tangles (NFT) containing hyperphosphorylated tau protein (109). In a microglia-depleted AD model, diffuse-like plaques increased, and compact-like plaques decreased, leading to enhanced neuritic dystrophy (110). However, repopulated microglia reverse this phenomenon and promote the transition from diffuse- to compact-like plaques, thereby causing compact-like plaques to dominate the microglia-repopulated regions, consequently limiting neuritic dystrophy (110). Of note, cortical microglia did not fully repopulate, possibly because of the marked heterogeneity in the microglial gene expression profile (111, 112). In addition, another study showed that microglial repopulation can limit the growth of amyloid plaques and significantly reduce the dendritic abnormalities caused by Aβ deposition (82). PD is characterized by loss of dopaminergic neurons and accumulation of intraneural Lewy bodies (113). The role of repopulated microglia in PD has recently attracted attention. In a mouse model with completely repopulated microglia followed by PLX3397 removal and 1-methyl-4-phenyl-1,2,3,6-tetrahydropyridine (MPTP) challenge, the repopulated microglia protect dopaminergic neurons and improve motility. However, among PLX3397-fed mice with drug cessation at the time of MPTP challenge, the loss of dopaminergic neurons was not significantly different between the repopulated microglia group and control group, indicating that the effects of repopulated microglia depend on the time point of PLX3397 cessation. Interestingly, in both models, the expression of neurotrophic factors and phagocytosis-related molecules was increased in the repopulated microglia group, but the expression of inflammation-related molecules was not affected (114). In brief, microglial replenishment is neuroprotective in PD mice and the currently available sparse information suggests potential use of repopulated microglia for PD treatment.

However, some studies have failed to show the positive effects of repopulated microglia. For example, Gratuze et al. (115) showed that although the repopulated microglia cluster around the plaque, similar to normal microglia, repopulated microglia significantly increased the homeostatic gene expression and failed to switch to the DAM phenotype, resulting in a marked increase in the seeding and spread of NP-tau. Thus, the repopulated microglia may not effectively reduce plaque-related toxicity.

### Microglial repopulation therapy for CNS trauma

Microglial activation is an essential innate immune response that protects the brain after trauma; however, a dysregulated immune response can lead to secondary damage (116). With the use of CSF1R inhibitors to treat brain trauma, repopulated microglia appear 3 days after drug withdrawal and, by 7 days, the number of microglia is higher than that in control mice. By 21 days, the number and morphology of the repopulated microglia were similar to the control group, albeit with smaller somas and longer and finer processes. Brain trauma leads to elevated levels of inflammatory factors. The repopulated microglia suppress lesion-induced inflammation, but do not possess the activation state of the original microglia, thereby making them less reactive. These results suggest that chronic inflammation is inherent with the microglia and not completely dependent on the environment (92). Therefore, the repopulated microglia can replace the activated microglia to prevent microglia-mediated chronic inflammation, which is an effective strategy to repair CNS trauma. Rice et al. (92) treated brain-injured mice with PLX3397 for 14 days followed by PLX3397 withdrawal, and found that the microglia repopulated the whole brain; the newly produced repopulated microglia had a naïve state morphology. Importantly, protracted inflammation was resolved by the microglial depletion and repopulation treatment. Ma et al. (25) also found that microglial depletion and repopulation in combination with gelatin hydrogel transplantation resolved acute and chronic inflammation, promoted endogenous neural stem/progenitor cell migration and neurogenesis, and improved the electrophysiological and functional recovery in mice with complete transection SCI. Notably, the repopulated microglia had no significant effect on trauma-induced astrocyte expression and reactivity (74, 92). Microglial depletion and repopulation also prevent radiation-induced hippocampal-dependent memory defects and loss of hippocampal PSD-95 (117). The increase in PSD-95 and synaptophysin puncta suggest that the repopulated microglia determine and regulate the synaptic landscape (92, 118). In a mouse model of brain injury, repopulated microglia attenuate learning deficits, stimulate neurogenesis, induce IL-6 production from neurons, and mediate neuroprotection. These neuroprotective and pro-regenerative microglia have a unique transcriptional profile and modulate the microenvironment, in particular, suppressing neurotoxic A1 astrocyte formation (118). In addition, the reactivity of repopulated microglia is reduced, the proliferative state is enhanced, and the gene expression levels of wound healing and repair are increased. Remarkably, the neuroprotective effect of repopulated microglia depends on the appropriate timing of treatment, and these cells are effective for the acute rather than post-acute phase of injury (118).

### Microglial repopulation therapy for aging brain

Compared to the adult brain, microglia in the aging brain are dysfunctional, with increased numbers, became “dystrophy”, decreased motility, altered signaling, impaired phagocytosis and proteostasis, and a greater pro-inflammatory profile (119, 120). Aging is characterized by increased expression of CD68+ and deposition of lipofuscin (121). With microglial regeneration, the expression of CD68+ in aged microglia is normalized to the adult level and the lipofuscin level is reduced (122). In addition, the densities and morphologies of repopulated microglia are restored to the adult levels, and these cells improve cognition in aged mice, increase the neuronal regeneration rates, alter the dendritic spine densities and neuronal complexities, and restore the neuronal physiological processes in the aging brain (123). However, a recent study reported that microglial repopulation does not improve synaptic transmission or cognitive function in aged animals (124). Furthermore, the repopulated microglia only partially affect the age-related mRNA signaling and do not sufficiently modify the neuroinflammatory immune responses, suggesting that responses to the inflammatory stimuli depend on the aging microenvironment rather than microglial state (122). Taken together, the aforementioned findings suggest that microglial replacement may be potentially useful to normalize changes in the aging brain. However, additional methods to treat neuroinflammation in the aging brain need to be explored.

### Microglial repopulation therapy for short-term memory and cognitive deficits

Microglia play integral roles in regulating neuronal activity and synaptic transmission (125), and microglia-neuron communication is critical for learning and memory in the adult brain (126). In addition, repopulated microglia after microglial depletion enhance short-term memory, which may be related to increased astrocyte density (127). The possible mechanisms underlying astrocyte involvement in memory formation include regulation of synaptic formation, transmission, and plasticity (127). Galactic cosmic ray (GCR) exposure can affect neuronal and microglial functions (128), and microglial depletion can improve radiation-induced memory deficits and cognitive dysfunction (129, 130). Following CSF1R inhibitors treatment, the repopulated microglia reduce lysosome-associated membrane protein 1 (LAMP-1) level, limit the GRC-induced phagocytic phenotype, and thus ameliorate cognitive deficits and synaptic loss. In addition, inflammatory chemokines, cytokines, and complement component 5a receptor (C5aR) expression levels are also reduced (128, 131). In line with this, previous studies have reported that C5aR antagonist treatment can improve cognitive performance in animal models of different diseases (132, 133).

### Microglial repopulation therapy for other diseases and deficits

Microglial repopulation therapy has also been used for the treatment of other diseases or deficits. For example, repopulated microglia did not prevent repeated social defeat (RSD)-induced stress sensitization and excessive immune and behavioral responses in mice, probably because multiple CNS cell types contribute to RSD sensitization, among which microglial priming is only partly involved. However, repopulated microglia effectively attenuated the immune and neuroinflammatory responses caused by LPS challenge following acute RSD (134). In a chronic neuroinflammation model (e.g., alcohol use disorders, AUDs), microglial depletion and repopulation can reverse chronic neuroimmune activation by normalizing proinflammatory cytokine levels and increasing protective trophic factors (135). Microglial depletion and repopulation did not affect the neuropathology of EAE or CNS T-cell responses (136). The repopulated microglia correct the anatomical defects, partially restore the auditory function (137), and eradicate fear memory in mice after fear conditioning (138). A recent study showed that repopulated microglia can reduce neuroinflammation, neurological deficits, and brain edema following intracerebral hemorrhage in the aged brain (139). These observations suggest that microglial repopulation therapy may be promising in the treatment of neurological diseases and deficits.

## Conclusions

However, several important problems need to be addressed in the future. First, CSF1R inhibitors not only depletes microglia but also affects peripheral myeloid cells, for example, PLX3397 reduces the specific subsets of circulating monocytes in a mouse AD model (140). Furthermore, off-target effects may occur with peripheral administration in genetic models (141). Therefore, approaches to deplete microglia alone need to be developed in the future. Second, the optimal timing of microglial depletion and repopulation in specific disease models need to be determined. As we mentioned previously, the initial 3 days after TBI are critical, during which microglial regeneration may play a neuroprotective role (141). The optimal treatment window period for other neurological diseases and deficits needs to be determined in future studies. Third, the current results are mainly based on rodent models, and the effects of microglial repopulation therapy in primates and humans are not clear. In the future, patients with CNS diseases or deficits may be treated with microglial repopulation therapy to promote functional recovery; however, currently, there are significant challenges to the use of this therapy.

## Author contributions

HF and TY contributed to the conception and design of the review. JZ and ZS contributed to searching information or preparing the figures or table. WS, YZ, YX, HF, and TY contributed to drafting and revising of manuscript. All authors contributed to the article and approved the final manuscript.

## Funding

This work was supported by National Natural Science Foundation of China (NO. 82102558; NO.31872310), Shandong Province Natural Science Foundation (ZR2020QH117), and China Postdoctoral Science Foundation (2021M691690).

## Conflict of interest

The authors declare that the research was conducted in the absence of any commercial or financial relationships that could be construed as a potential conflict of interest.

## Publisher’s note

All claims expressed in this article are solely those of the authors and do not necessarily represent those of their affiliated organizations, or those of the publisher, the editors and the reviewers. Any product that may be evaluated in this article, or claim that may be made by its manufacturer, is not guaranteed or endorsed by the publisher.
